# Serum selenium accelerates the development of metabolic disorders in a metabolically healthy obese U.S. population: a retrospective cross-sectional analysis of a population-based study from the NHANES (2011-2018)

**DOI:** 10.3389/fimmu.2024.1398299

**Published:** 2024-08-29

**Authors:** Bei Li, Jieli Chen, Haiyan Ma, Ying Yu, Shengnan He, Lan Yang

**Affiliations:** ^1^ Department of Gastrointestinal Surgery, Peking University Shenzhen Hospital, Shenzhen, China; ^2^ Department of Urology, Shenzhen Children’s Hospital, Shenzhen, China; ^3^ Department of Breast and Thyroid Surgery of Shenzhen Second People’s Hospital, The First Affiliated Hospital of Shenzhen University, Health Science Center, Shenzhen Second People’s Hospital, Shenzhen, China; ^4^ Department of Gastroenterology of Shenzhen Second People’s Hospital, The First Affiliated Hospital of Shenzhen University, Health Science Center, Shenzhen Second People’s Hospital, Shenzhen, China

**Keywords:** serum selenium, metabolically unhealthy obesity (MUO), body mass index (BMI), national health and nutrition examination survey (NHANES), obesity

## Abstract

**Background:**

Obesity represents a significant risk factor for the development of metabolic abnormalities. However, it is not inevitable that all individuals with obesity will develop these disorders. Selenium has been demonstrated to play a role in maintaining metabolic homeostasis *in vivo*, with the ability to regulate relevant signaling pathways involved in glucose and lipid metabolism processes. Previous studies have indicated that selenium concentrations in obese individuals are higher than those reported in the general population. These findings the question of whether altered selenium concentrations may act as important triggers for accelerating metabolic imbalances in the obese population. The aim of this study was to examine the potential correlation between serum selenium concentrations and the risk of developing metabolic abnormalities in individuals with obesity.

**Methods:**

The present study included 6,125 participants from the 2011-2018 National Health and Nutrition Examination Survey (NHANES) who were aged between 20 and 80 years, with a body mass index (BMI) of 30 kg/m^2^ or greater, and met the inclusion and exclusion criteria. Weighted generalized linear regression analyses were conducted to evaluate the associations between serum selenium concentrations and the conversion of metabolically healthy obesity (MHO) to metabolically unhealthy obesity (MUO). A generalized additive model (GAM) and a two-piecewise linear regression model were employed to investigate the saturation threshold effect between selenium and MUO. The correlation between different selenium concentration intervals and metabolic diseases was evaluated by categorizing selenium concentrations according to the saturation threshold. Furthermore, this study investigated the correlation between serum selenium and lipid concentrations in obese females and between serum selenium and blood pressure in obese males.

**Results:**

The weighted prevalence of MUO in the study population was 48.35%. After rigorous adjustment for sociodemographic, physical, and laboratory test covariates, the weighted odds ratio (OR) of MUO increased by 44% for every 1 µM increase (approximately 78.74 µg) in the serum selenium concentration (weighted OR=1.44; 95% CI=1.09 - 1.91; *P*=0.018). Second, GAM analysis and saturation threshold analyses revealed an inverted U-shaped relationship between serum selenium and metabolic abnormalities in males, with a corresponding inflection point (K) of 2.82 µM. When the serum selenium concentration was below the K-value, the effects of serum selenium were mainly on blood pressure, especially diastolic blood pressure (DBP) (weighted β: 3.34; 95% CI= 0.25 - 6.44; *P*=0.038). Conversely, the correlation between the serum selenium concentrations and metabolic homeostasis imbalance in females was linear. When the selenium concentration exceeded 2.12 µM, the increase in selenium content was accompanied by increases in total cholesterol (TC, weighted β=0.54, 95% CI=0.32 - 0.76; *P*=0.000) and triglyceride (TG, weighted β=0.51, 95% CI=0.27 - 0.75; *P*=0.000) concentrations.

**Conclusions:**

The findings of our study indicate that selenium supplementation strategies for individuals with obesity should be tailored to the sex of the individual. In females, serum selenium concentration above the saturation threshold primarily facilitates the transition from MHO to MUO by influencing alterations in serum lipid metabolism. Maintaining selenium concentrations below the threshold levels is highly important for preventing the conversion of MHO to MUO. In males, serum selenium concentrations above the threshold were found to be effective in preventing an elevation in blood pressure, particularly in improving systolic blood pressure (SBP). Nevertheless, serum selenium concentrations below the threshold are linked to an increased risk of hypertension in obese individuals, particularly those with elevated diastolic blood pressure (DBP). Further research is needed to elucidate the optimal serum selenium concentration that exerts deleterious effects on blood pressure.

## 1 Introduction

Obesity is a significant risk factor for metabolic diseases, which can include insulin resistance, impaired fasting glucose and/or tolerance, dyslipidemia and hypertension (1). However, not all obese individuals have metabolic syndrome (MetS) or insulin resistance. Epidemiological studies indicate that approximately one-third of individuals classified as obese do not display significant abnormalities in serum glucose, serum lipids, blood pressure, insulin resistance or inflammation. This condition is known as metabolically healthy obesity (MHO) (2). Compared with individuals with metabolically unhealthy obesity (MUO), those with MHO exhibit relatively stable physical activity status, nutritional status, ectopic fat, and visceral adiposity (3). MHO individuals are often considered to have a transitional phenotype. The incidence of MHO declines progressively over time. In addition, the prevalence of metabolic disease is associated with the severity and duration of obesity (4). However, the factors that contribute to the conversion of MHO to MUO are unclear.

Obesity was defined as having a body mass index (BMI) ≥ 30 kg/m^2^. Although BMI moderately correlates with direct measures of body fat content, it does not directly measure the amount of adiposity. Importantly, BMI is not an accurate predictor of cardiometabolic risk and is not a measure of total body fat or abdominal fat accumulation (5, 6). Furthermore, obesity is associated with a wide range of comorbidities and health risk factors, even within the same BMI range (6). Research has demonstrated a strong correlation between the number and severity of metabolic abnormalities and the risk of adverse outcomes (7). Approximately 30-50% of MHO individuals become MUO individuals within 4-20 years (8, 9). Individuals with MUO have a significantly greater risk of type 2 diabetes, coronary heart disease (CHD), and all-cause mortality than MHO individuals (10, 11).

Recently, there has been a notable increase in interest in essential trace elements and minerals, despite their relatively minor proportion of less than 0.01% of total body weight. Selenium is a naturally occurring chemical element that can be found in soil, water, and air; it then enters the food chain and is absorbed by plants, animals, and humans. The principal sources of selenium in the human diet are cereals, meat, poultry, fish, and eggs (12). The primary chemical form of selenium present in food is selenomethionine (SeMet). In the liver, SeMet is metabolized to selenide, which is subsequently utilized in the synthesis of selenoproteins that are involved in a variety of biological functions, including antioxidant stress, anti-inflammatory, lipid metabolism, and immunomodulation (13, 14). Selenium intake is essential for the proper function of the cardiovascular, endocrine, nervous and immune systems. Therefore, some medical guidelines advise that individuals consume selenium supplements on a daily basis to protect against free radical-induced cellular damage (15, 16). Notably, the range of beneficial doses of selenium is more limited. Insufficient and excessive intake of selenium can have adverse health effects. For example, selenium plays a regulatory role in insulin signaling pathways, which may influence carbohydrate and lipid metabolism (13, 17). Consequently, when excess selenium is consumed, there is an increased risk of developing metabolic diseases such as coronary heart disease (CHD), diabetes mellitus (DM), and metabolic dysfunction-associated with fatty liver disease (MAFLD) (14, 18–20).

Despite extensive research on the relationship between serum selenium and DM in previous investigations, the relationship between serum selenium and obesity has primarily been examined with respect to the capacity to of selenium modulate adipocyte biology. Several animal studies have shown that the expression of selenoprotein in the adipose tissue of obese mice is markedly elevated compared with that in healthy mice. The metabolism of selenium and selenoproteins may be relevant to adipocyte physiology and may play a role in the pathogenesis of obesity. The precise relationship between elevated serum selenium concentrations *in vivo* and the conversion of MHO to MUO remains unclear. The type of metabolic dysregulation in individuals who obese is associated with higher serum selenium concentrations has also yet to be elucidated.

Given the high prevalence of obesity and the associated health risks, in the present study, we conducted a cross-sectional study using a representative sample of U.S. adults from the 2011 to 2018 National Health and Nutrition Examination Survey (NHANES) to assess whether there were differences in selenium concentrations between the MUO and MHO groups. The aim of this study was not only to elucidate the association between serum selenium concentrations and the risk of metabolic homeostasis imbalance, but also to identify the metabolic role of selenium and to ascertain the selenium-related changes in metabolic indicators that occur during the transition from MHO to MUO.

## 2 Methods

### 2.1 Data source and study population

We conducted a secondary scientific analysis of the NHANES database from 2011-2018, The NHANES database is an independent cross-sectional survey conducted by the National Center for Health Statistics (NCHS) of the Centers for Disease Control and Prevention (CDC) that assesses the health and nutritional status of a nationally representative sample of U.S. civilian noninstitutionalized individuals from the general population. Details of the survey in terms of its planning, conduct, and design are available on the official website (https://www.cdc.gov/nchs/nhanes/about_nhanes.htm). The survey included questionnaires; physical examinations; household interviews that covered demographic, dietary, and health-related questions and examinations; and laboratory tests (21, 22).

**Figure 1:**
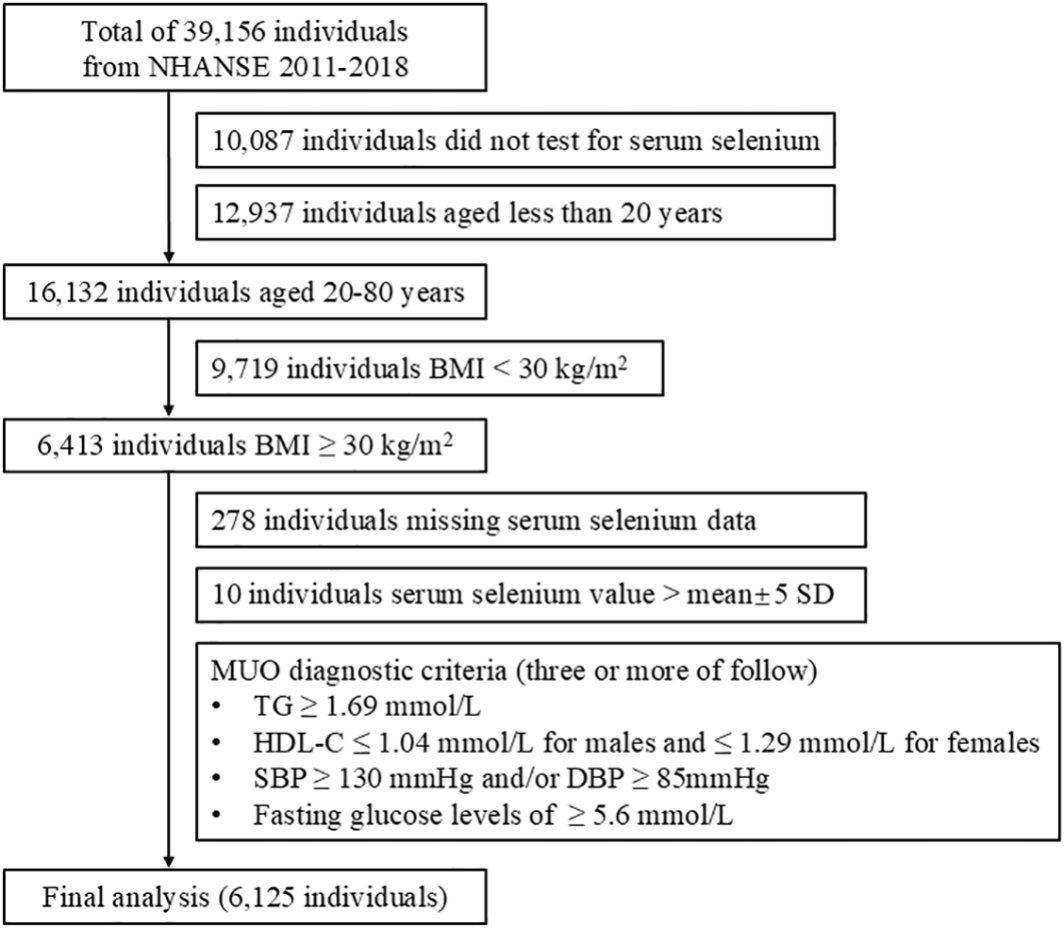
Flow chart of screening and enrollment of study participants.

A total of 39,156 participants were included in the NHANES for the period between 2011 and 2018. After the exclusion of 10,087 participants who did not have serum selenium data, 12,937 participants younger than 20 years of age, 9,719 participants with a BMI less than 30 kg/m^2^, 278 participants with missing serum selenium data, and 10 participants with selenium concentrations that did not fall within the mean±5 standard deviation (SD) range, the final analysis included 6,125 participants. The screening process for the study participants is illustrated in **Figure 1**.

### 2.2 Measurement of serum selenium concentrations

Serum samples were processed, stored, and shipped to the Division of Laboratory Sciences at the National Center for Environmental Health and the CDC for analysis. Serum selenium concentrations were quantified via inductively coupled plasma-mass spectrometry. The lower limit of detection for serum selenium was 0.311 μM for the NHANES 2011-2018. All included populations presented serum selenium values above the detection limit. Detailed instructions for sample collection, processing, and quality assessment are available on the NHANES website:(https://wwwn.cdc.gov/Nchs/Nhanes/2011-2012/PBCD_G.htm; https://wwwn.cdc.gov/Nchs/Nhanes/2013-2014/PBCD_H.htm; https://wwwn.cdc.gov/Nchs/Nhanes/2015-2016/PBCD_I.htm; https://wwwn.cdc.gov/Nchs/Nhanes/2017-2018/PBCD_J.htm).

### 2.3 Assessment

Demographic data, including age, sex, race (non-Hispanic white, non-Hispanic black, non-Hispanic Asian, Mexican American, and other races), marital status (married and living with partner; widowed, divorced, and separated; never married), education (college and above, high school, less than high school), family size (1-3 people, 4-6 people and more than 7 people) and family income (<$45,000, $45,000-$99,999, and ≥ $1,00,000), were collected from all participants. The following physical examination data were collected: standing height (cm), weight (kg), body mass index (BMI, kg/m^2^), waist circumference (cm), systolic blood pressure (SBP, mmHg) and diastolic blood pressure (DBP, mmHg). Additionally, laboratory data including blood glucose (mM), glycohemoglobin (HBA1C, %), triglyceride (TG, mM), total cholesterol (TC, mM), high-density lipoprotein cholesterol (HDL-C, mM) and low-density lipoprotein cholesterol (LDL-C, mM), were obtained.

A questionnaire was used to collect data regarding the smoking history of the participants, who were classified into three categories: never, former, and current smokers. Additionally, the participants were queried regarding their alcohol consumption status (never, ever, and current), with responses from current drinkers converted to reflect their weekly alcohol intake. The final exposure categories were as follows: never, former, mild (defined as 1 time/week), moderate (1-3 times/week), and vigorous (> 3 times/week). The term “physical activity (PA)” encompasses both work and recreation activities. This definition is consistent with that presented in a previous report (23). In brief, the level of PA is determined by the PA Questionnaire in the NHANES, which is based on metabolic equivalent (MET) values, the type of activity, the frequency of engagement in the activity weekly, and the duration of the activity. The PA score was calculated via the following formula: PA=MET×weekly frequency×duration of each physical activity. Participants with a PA score of 0 were classified as exhibiting no PA, whereas those with a PA score of ≥ 1 were classified as engaging in some PAs.

### 2.4 Diagnostic criteria for MUO

Individuals meeting two or fewer of the following diagnostic criteria were classified as having MUO: BMI of 30 kg/m^2^ or higher; triglyceride≥1.69 mmol/L; HDL-C ≤ 1.04 mmol/L for males and ≤ 1.29 mmol/L for females; blood pressure (systolic BP) ≥ 130 mmHg and/or diastolic BP ≥ 85 mmHg; and fasting glucose levels ≥ 5.6 mmol/L. MUO was diagnosed if three or more of the above diagnostic criteria were met (24). Participants were identified as having selected components of metabolic syndrome if they were taking medication to modify blood pressure or lipid or carbohydrate metabolism. This information was obtained from the participants’ questionnaire responses.

### 2.5 Statistical analysis

Appropriate weighting methods are employed to account for the complex sampling design, thereby ensuring the provision of nationally representative results in accordance with the guidelines set forth by the NHANES (25). The basic characteristics of categorical variables are expressed as counts and percentages (%), and the basic characteristics of continuous variables are described by weighted means and SDs. The differences between continuous variables were analyzed via weighted linear regression models, whereas the differences between categorical variables were analyzed via weighted chi-square tests.

The objective of this study was to evaluate the associations between serum selenium concentrations and MUO while controlling for confounding covariates. To ascertain the correlation between the serum selenium concentration and the incidence of MUO, a univariate logistic regression model was used for the preliminary analysis. Model 1 was adjusted for sociodemographic variables, including age, race, sex, marital status, family size, education and family income. Model 2 was adjusted for blood glucose, HBA1C, SBP, DBP, TC, TG, HDL-C, BMI, waist circumference, physical activity, smoke and alcohol use. Model 3 adjusts for both the covariates present in Model 2 and Model 1. These confounders were selected on basis of their associations with the outcomes of interest or a change in effect estimate of more than 10%.

In addition, smooth curves were fitted via a generalized additive model (GAM). A log-likelihood ratio test was conducted to compare the single-line model with the two-piecewise linear regression model to determine the existence of a saturation threshold. The inflection point (K), which connects the line segments, was identified as the maximum likelihood value calculated via the recursive algorithm method. Furthermore, the risk of serum selenium concentrations and MUO occurring on either side of the inflection point was also reassessed via weighted generalized linear modeling.

The statistical analyses were conducted using R (http://www.R-project.org) and Empower Stats (http://www.empowerstats.com, X&Y Solutions, Inc., Boston, MA). A two-tailed *P* value less than 0.05 was considered to indicate statistical significance.

### 2.6 Research ethics

The NHANES study protocols used were approved by the Research Ethics Review Board of the NCHS. The methods were conducted in accordance with the Strengthening the Reporting of Observational Studies in Epidemiology (STROBE) statement. Written informed consent was obtained from all study participants.

## 3 Results

### 3.1 Baseline characteristics of the participants

The final cohort consisted of 6,125 participants, selected on the basis of the pre-established inclusion and exclusion criteria, from a total of 39,156 participants in NHANES from 2011-2018. The participants were classified into two groups on the basis of their metabolic health status. The characteristics of the study population are detailed in **Supplementary Table 1**. The weighted prevalence of MUO was 48.35%, with 52.24% in males and 47.76% in females. The present study indicated that participants susceptible to MUO were more likely to be male and non-Hispanic White. Additionally, these participants may have been living in households with small populations, potentially due to circumferences such as widowed, divorced, or separated. They had a history of smoking, lower alcohol consumption, and lower levels of physical activity. Furthermore, the MUO group presented elevated waist circumference, blood glucose, HBAIC, TC, TG, HDL-C, LDL-C and blood pressure, which were significantly greater than those of the MHO group (all *P*<0.05).

The present study demonstrated the observed discrepancies in the serum selenium concentrations observed among the participants in the MHO and MUO groups. **Supplementary Table 1** shows that the weighted mean serum selenium concentration in participants with MUO was 2.48 µM (approximately 195 µg/L), which was significantly higher than that observed in the MHO group (*P*=0.006). The weighted prevalence of MUO was subsequently examined on the basis of tertiles of serum selenium concentrations (T1: < 2.30 µM; T2: 2.31 - 2.55 µM; T3: 2.56 - 4.09 µM). As illustrated in **Table 1**, there was a positive correlation between the weighted prevalence of MUO and increasing selenium concentrations. Specifically, the weighted prevalence was 44.39% in the T1 group and increased to 51.38% in the T3 group (*P*=0.0094). Notably, participants in the T3 group presented an elevated risk of hyperlipidemia (*P*=0.001) and DM (*P*=0.023) than did those in the MHO group.

**Table 1:** Weighted characteristics of the study participants based on the serum selenium tertile.

Characteristic	Serum selenium (Se) tertiles			*P* value
	T1 (Se:≤2.30 µM)	T2 (Se: 2.31-2.55 µM)	T3 (Se:2.56-4.09 µM)	
**Age** (Years)	49.32 (48.28, 50.36)	48.03 (47.09, 48.97)	48.62 (47.50, 49.73)	0.172
Sex (%)				<0.001
Male	40.27 (36.88, 43.76)	45.62 (42.53, 48.75)	52.56 (49.12, 55.98)	
Female	59.73 (56.24, 63.12)	54.38 (51.25, 57.47)	7.44 (44.02, 50.88)	
Race/Ethnicity (%)				<0.001
Non-Hispanic White	58.79 (53.75, 63.64)	64.42 (59.84, 68.75)	65.74 (60.52, 70.59)	
Non-Hispanic Black	18.21 (14.93, 22.01)	13.16 (10.67, 16.12)	11.34 (8.98, 14.23)	
Non-Hispanic Asian	1.69 (1.24, 2.31)	2.23 (1.66, 3.00)	2.16 (1.58, 2.95)	
Mexican American	9.83 (7.48, 12.81)	11.24 (8.80, 14.24)	10.67 (8.18, 13.81)	
Others	11.48 (9.47, 13.86)	8.95 (7.39, 10.80)	10.09 (8.40, 12.08)	
Marital Status (%)				0.016
Married/Living with partner	59.47 (56.08, 62.77)	62.29 (58.76, 65.70)	64.19 (60.99, 67.26)	
Widowed/ Divorced/Separated	23.17 (21.02, 25.47)	21.41 (19.05, 23.98)	17.67 (15.45, 20.14)	
Never married	17.33 (14.67, 20.37)	16.29 (13.76, 19.18)	18.10 (15.64, 20.86)	
Education (%)				0.001
Collage and above	55.22 (51.85, 58.55)	61.07 (58.63, 63.47)	62.55 (58.30, 66.60)	
High school	27.62 (25.29, 30.07)	25.09 (22.80, 27.52)	24.96 (21.78, 28.43)	
Less than high school	17.09 (14.74, 19.73)	13.81 (12.05, 15.79)	12.47 (10.60, 14.61)	
Family Size (%)				0.799
1-3 people	66.38 (62.98, 69.62)	66.81 (64.05, 69.47)	68.17 (64.94, 71.23)	
4-6 people	30.59 (27.87, 33.46)	30.54 (28.02, 33.19)	28.82 (25.90, 31.94)	
More than 7 people	3.03 (1.91, 4.76)	2.64 (2.06, 3.38)	3.01 (2.35, 3.85)	
Family income (%)				0.242
< $45,000	47.14 (43.65, 50.66)	43.18 (40.41, 45.99)	42.54 (39.12, 46.02)	
$45,000-$99,999	28.73 (25.16, 32.59)	33.13 (29.48, 37.00)	32.51 (29.24, 35.97)	
≥$100,000	19.07 (16.46, 21.98)	19.98 (16.86, 23.52)	19.88 (16.47, 23.80)	
Smoking (%)				0.285
Never	56.02 (52.67, 59.32)	56.25 (52.42, 60.00)	54.77 (52.12, 57.39)	
Former	26.61 (23.54, 29.92)	26.44 (23.16, 30.00)	29.71 (27.39, 32.13)	
Now	17.37 (14.86, 20.20)	17.29 (15.39, 19.37)	15.46 (13.41, 17.76)	
Alcohol user (%)				0.005
Never	11.16 (9.42, 13.18)	8.27 (6.97, 9.79)	7.18 (6.22, 8.27)	
Former	11.05 (9.18, 13.24)	10.12 (8.26, 12.35)	9.65 (7.89, 11.75)	
Mild	30.44 (27.52, 33.53)	31.88 (29.34, 34.53)	35.18 (32.05, 38.44)	
Moderate	13.85 (12.19, 15.70)	17.41 (15.06, 20.04)	16.51 (14.03, 19.33)	
Heavy	19.54 (17.38, 21.90)	20.23 (17.84, 22.85)	20.58 (18.04, 23.36)	
Work Activity (%)				0.219
No	55.02 (51.90, 58.10)	52.77 (49.57, 55.96)	51.20 (47.61, 54.77)	
Yes	44.98 (41.90, 48.10)	47.23 (44.04, 50.43)	48.80 (45.23, 52.39)	
Recreational Activity (%)				0.400
No	55.49 (51.83, 59.09)	53.11 (49.73, 56.45)	52.38 (48.61, 56.12)	
Yes	44.51 (40.91, 48.17)	46.89 (43.55, 50.27)	47.62 (43.88, 51.39)	
BMI (kg/m^2^)	36.60 (36.20, 37.00)	35.97 (35.59, 36.35)	35.93 (35.53, 36.33)	0.015
Height (cm)	166.9 (166.3,167.5)	168.0 (167.4,168.6)	168.8 (168.0, 169.5)	0.001
Weight (kg)	102.1 (101.9,103.3)	101.6 (100.6,102.6)	102.6 (101.1, 104.0)	0.615
Waist circumstance (cm)	115.5 (114.5, 116.5)	114.5 (113.7, 115.3)	115.7 (114.7, 116.6)	0.166
Glucose (mM)	5.80 (5.68, 5.92)	5.86 (5.74, 5.97)	6.06 (5.92, 6.21)	0.027
HBA1C (%)	5.85 (5.79, 5.90)	5.82 (5.76, 5.88)	5.96 (5.89, 6.03)	0.012
TC (mM)	4.81 (4.73, 4.89)	4.95 (4.89, 5.02)	5.14 (5.08, 5.20)	<0.001
TG (mM)	1.75 (1.66, 1.83)	1.99 (1.89, 2.08)	2.19 (2.10, 2.27)	<0.001
HDL-C (mM)	1.28 (1.26, 1.31)	1.25 (1.23, 1.27)	1.23 (1.21, 1.26)	0.006
LDL-C (mM)	2.86 (2.76, 2.96)	2.99 (2.93, 3.05)	2.97 (2.89, 3.05)	0.147
SBP (mmHg)	126 (125, 127)	125 (125, 126)	126 (125, 127)	0.956
DBP (mmHg)	71 (71, 72)	73 (73, 74)	74 (73, 75)	<0.001
MUO				0.009
No	55.61 (52.30, 58.88)	51.49 (47.77, 55.20)	48.62 (46.01, 51.24)	
Yes	44.39 (41.12, 47.70)	48.51 (44.80, 52.23)	51.38 (48.76, 53.99)	

Data in the table: For continuous variables: survey-weighted means (95% CI), and P-value were obtained via survey-weighted linear regression (svyglm); For categorical variables: survey-weighted percentages (95% CI), and P-value was by survey-weighted chi-square test (svytable).

Body mass index, BMI; Glycohemoglobin, HBA1C; Total Cholesterol, TC; Triglyceride, TG; HDL cholesterol, HDL-C; LDL cholesterol, LDL-C; Systolic blood pressure, SBP; Diastolic blood pressure, DBP, metabolically unhealthy obesity, MUO.

### 3.2 Serum selenium concentrations associated with the risk of developing MUO

Four weighted multiple regression models were used to assess the correlation between the serum selenium concentration and the likelihood of MHO progressing to MUO. The results revealed a statistically significant correlation between the serum selenium concentration and the risk of MHO developing into MUO in all the models, including the crude model and the models adjusted for differences in covariates (**Table 2**). The fully adjusted Model 3 revealed that the weighted odds ratio (OR) for MUO increased by 44% for each unit increase in serum selenium (OR=1.44, 95% CI=1.09 - 1.91; *P*=0.018). Furthermore, the associations between serum selenium concentrations and metabolic abnormalities in both sexes were examined. These results indicate that the influence of selenium on metabolic functions in individuals with obesity is not contingent on sex.

**Table 2:** Weighted generalized linear regression analysis for the association between serum selenium and MUO.

Selenium, µM	Crude model		Adjust model 1		Adjust model 2		Adjust model 3	
	OR (95% CI)	*P* value	OR (95% CI)	*P* value	OR (95% CI)	*P* value	OR (95% CI)	*P* value
Per 1 µM increase	1.43 (1.11, 1.82)	0.007	1.40 (1.09, 1.81)	0.012	1.45 (1.12, 1.89)	0.008	1.44 (1.09, 1.91)	0.018
Sex								
Male	1.07 (0.75, 1.53)	0.701	1.11 (0.77, 1.59)	0.583	1.52 (1.02, 2.27)	0.044	1.52 (1.03, 2.25)	0.046
Female	1.62 (1.19, 2.21)	0.004	1.76 (1.30, 2.39)	0.001	1.40 (0.97, 2.02)	0.083	1.53 (1.05, 2.25)	0.030

Result in table: Weighted OR (95% CI) P value.

Outcome: MUO.

Exposure: Serum selenium.

Non-adjusted model adjust for: none.

Adjust I model adjust for: age, race, sex, marital status, family size, education and family income.

Adjust II model adjust for: glucose; HBA1C, SBP, DBP, TC, TG, HDL.C, BMI, waist (cm), work activity, recreational activity, smoke and alcohol use.

Adjust III model adjust for: age, race, sex, marital status, family size, education, family income, glucose, HBA1C, SBP, DBP, TC, TG, HDL-C, BMI,

Waist circumference (cm), work activity, recreational activity, smoke and alcohol use.

### 3.3 Characterization of the correlation between serum selenium concentrations and metabolic abnormalities in obese individuals

**Figure 2:**
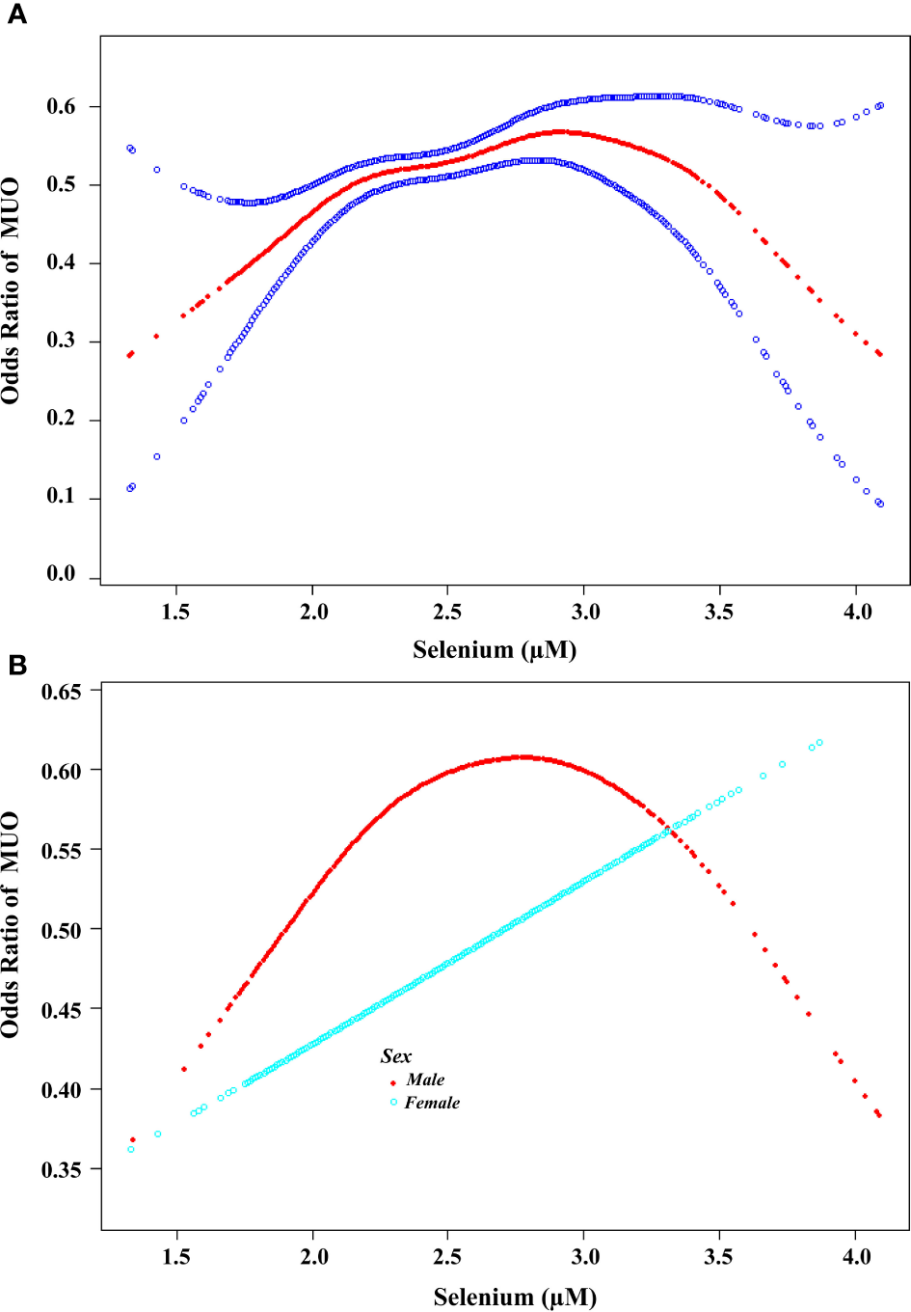
Association between the serum selenium concentration and the risk of developing MUO. **(A)** A threshold nonlinear association between serum selenium concentration and the risk of developing MUO was found via the GAM (*P*<0.05). The red line represents the smooth curve fit between the variables. The blue bands represent the 95% confidence intervals (CIs) of the fits. All the analyses were adjusted for Adjust for: age, sex, race, marital status, family size, education level, family income, glucose, HBA1C, SBP, DBP, TC, TG, HDL-C, BMI, waist circumference(cm), work activity, recreational activity, smoke and alcohol use. **(B).** Association between the serum selenium concentration and the risk of developing MUO according to sex. A threshold, nonlinear association between the serum selenium concentration and MUO was found via a GAM (*P*<0.05). The red line represents the smooth curve for males. The blue line represents the smooth curve of females. All the analyses were adjusted for age, race, marital status, family size, education level, family income, glucose, HBA1C, SBP, DBP, TC, TG, HDL-C, BMI, waist circumference(cm), work activity, recreational activity, smoke and alcohol use.

A log-likelihood ratio test was conducted to compare the nonsegment model to the segmented regression model, which was used to determine the threshold. The results of the log-likelihood ratio test indicate that there is a segmental relationship between the serum selenium concentration and MUO. The recursive algorithm was used to calculate the inflection point (K), which was determined to be 2.81 µM (approximately 222 µg/L) (**Figure 2A**).

Further examination was conducted to investigate the association between serum selenium concentrations and the risk of MHO developing into MUO by sex. In males, the effect size was 1.64, with a 95% CI of 1.09-2.48 and a *P* value of 0.018 on the left side of the K value (K=2.82). In females, the log-likelihood ratio of the two-segment linear model was 0.182, suggesting that the standard linear model provided a more accurate representation of the relationship between serum selenium and metabolic abnormalities (**Table 3**). As illustrated in **Figure 2B**, the correlation between serum selenium and MUO displayed a U-shaped pattern in males and a linear correlation in females.

**Table 3:** Threshold effect analysis of serum selenium concentrations on the risk developing MUO.

Characteristics	Male OR (95% CI), *P* value	Female OR (95% CI), *P* value
Fitting by standard linear model	1.16 (0.85, 1.59) 0.348	1.54 (1.13, 2.09) 0.006
Fitting by two-piecewise linear model		
Inflection point (K)	2.82	2.12
< K	1.64 (1.09, 2.48) 0.018	4.10 (0.93, 17.94) 0.061
> K	0.38 (0.15, 0.93) 0.035	1.35 (0.94, 1.94) 0.103
Log-likelihood ration	0.011	0.182

Result in table: OR (95% CI) P value.

Outcome: MUO.

Exposure: Serum selenium.

Adjust for: age, race, marital status, family size, education level, family income, glucose, HBA1C, SBP, DBP, TC, TG, HDL-C, BMI, waist circumference (cm), work activity, recreational activity, smoke and alcohol use.

### 3.4 Effects of the selenium threshold interval on the progression of MHO to MUO

Metabolic abnormalities such as hypertension, hyperlipidemia, DM, stroke, and CHD are associated with dysfunctional metabolic homeostasis. The present study revealed a significantly higher prevalence of hypertension, DM, stroke, and CHD in the MUO group compared to the MHO group. In contrast, no significant difference was observed in the prevalence of hyperlipidemia between the two groups (**Supplementary Table 2**). We subsequently examined the correlation between the selenium concentration interval and metabolism-related abnormalities on the basis of the saturation threshold. After adjusting for all potential confounding covariates, a statistically significant increased risk of developing hypertension was observed among participants with serum selenium concentrations less than 2.82 µM in males. **Table 4** illustrates the impact of different serum selenium threshold intervals on blood pressure in obese males. When the selenium concentration was less than 2.82 μM, the weighted β-value was -1.69 (95% CI: -5.51 - 2.13) for SBP and 3.34 (95% CI: 0.21 - 6.44) for DBP. The corresponding *P*-values were 0.389 and 0.038, respectively. During the concentration interval, a positive correlation was observed between selenium and DBP, with an increase of 3.34 mmHg in DBP for every 1 μM increase in serum selenium (**Table 5**). Conversely, when the selenium concentration exceeded 2.82 μM identified, which was negatively associated with SBP, a weighted β-value of -10.70 (95% CI: -17.83 - -3.57, *P*=0.005) was observed, along with a weighted β-value of -0.30 (95% CI: -6.25 - 5.66), with a *P* value of 0.923 for DBP. The results indicated a statistically significant inverse association between serum selenium concentrations and SBP, with a 10.70 mmHg reduction observed for each unit increase in serum selenium.

**Table 4:** Effect of metabolic abnormalities events on the serum selenium saturation threshold.

	Male (K=2.82)		Female (K=2.12)	
	Weighted OR (95% CI)	*P-*value	Weighted OR (95% CI)	*P-*value
Hypertension				
≤ K	1.93 (1.04, 3.59)	0.047	1.20 (0.21, 7.01)	0.831
> K	0.40 (0.10, 1.51)	0.188	0.85 (0.44, 1.66)	0.622
Hyperlipidemia				
≤ K	0.83 (0.42, 1.63)	0.567	0.11 (0.00, 2.53)	0.154
> K	1.89 (0.43, 8.33)	0.382	2.31 (1.17, 4.55)	0.017
DM				
≤ K	2.40 (0.89, 6.49)	0.082	2.38 (0.14, 40.91)	0.824
> K	1.21 (0.28, 5.18)	0.788	0.69 (0.35, 1.38)	0.303
CHD				
≤ K	0.86 (0.29, 2.59)	0.781	0.71 (0.01, 55.48)	0.872
> K	0.69 (0.07, 6.30)	0.729	1.56 (0.52, 4.67)	0.410
Stroke				
≤ K	0.62 (0.20, 1.97)	0.429	1.38 (0.05, 38.00)	0.842
> K	0.14 (0.01, 3.63)	0.250	0.86 (0.36, 2.07)	0.728

Model adjust for: age, race, marital status, family size, education level, family income, glucose, HBA1C, SBP, DBP, TC, TG, HDL-C, BMI, waist circumference (cm), work activity, recreational activity, smoke and alcohol use.

**Table 5:** The effect of higher serum selenium concentration (> 2.82µM) on blood pressure in males.

	Se ≤ 2.82 µM		Se > 2.82 µM	
	Weighted β (95% CI)	*P* value	Weighted β (95% CI)	*P* value
SBP	-1.69 (-5.51, 2.13)	0.389	-10.70 (-17.83, -3.57)	0.005
DBP	3.34 (0.21, 6.44)	0.038	-0.30 (-6.25, 5.66)	0.923

In females, a linear association was observed between the serum selenium concentration and the risk of developing metabolic abnormalities. An elevated risk of hyperlipidemia was observed in participants whose serum selenium concentrations exceeded 2.12 µM. **Table 6** illustrates the effects of different selenium threshold intervals on the serum lipid profiles of females. No statistically significant differences were detected in the effects of serum selenium on TG (weighted β=-0.78, 95% CI= -2.12 - -0.55; *P*=0.254), TC (weighted β=0.50, 95% CI= -0.45 -1.43; *P*=0.314), HDL-C (weighted β= -0.03, 95% CI= -0.42 - 0.36 *P*=0.881), or LDL-C (weighted β= 0.39, 95% CI= -0.75 - 1.53; *P*=0.508) at selenium concentrations below 2.12 μM. However, when the serum selenium concentration exceeded the threshold, we observed that the elevated serum TC (weighted β=0.54, 95% CI=0.32 - 0.76; *P*=0.000) and TG (weighted β=0.51, 95% CI=0.27 - 0.75; *P*=0.000) concentrations were elevated. At a serum concentration of 1 μM, the concentrations of TC and TG increased by 0.54 μM and 0.51 μM, respectively. However, no effects on HDL-C (weighted β= -0.00, 95% CI= -0.07 - 0.06; *P*=0.937) or LDL-C (weighted β=0.18, 95% CI= -0.07 - 0.43; *P*=0.159) concentrations were detected.

**Table 6:** The effect of higher serum selenium concentration (> 2.12µM) on serum lipids in females.

	Se ≤ 2.12 µM		Se > 2.12 µM	
	Weighted β (95% CI)	*P* value	Weighted β (95% CI)	*P* value
TC	0.49 (-0.45, 1.43)	0.314	0.54 (0.32, 0.76)	0.000
TG	-0.78 (-2.12, 0.55)	0.254	0.51 (0.27, 0.75)	0.000
HDL-C	-0.03 (-0.42, 0.36)	0.880	-0.00 (-0.07, 0.06)	0.937
LDL-C	0.39 (-0.75, 1.53)	0.508	0.18 (-0.07, 0.43)	0.159

## 4 Discussion

Dysmetabolism is defined as a pathological state of energy distribution and storage. These metabolic abnormalities are regulated by micronutrients that impact the structure of proteins, enzymes, and complex carbohydrates. Previous studies have indicated a potential correlation between selenium concentrations and the development of central obesity, hypertriglyceridemia, hyperglycemia, hyperglycemia, and hypertension (26). However, previous reports on the relationship between serum selenium concentrations and the risk of metabolic abnormalities are inconclusive. For example, a study of 13,289 participants revealed an inverse correlation between selenium levels and increasing BMI. Another cross-sectional study of a Chinese adult population revealed that serum selenium concentrations were positively associated with the risk of hyperglycemia and dyslipidemia (27). Furthermore, the IMMIDET study, which was adjusted for multiple confounders, confirmed a direct association between metabolic syndrome and high serum selenium concentrations (28). The findings of the present study indicate that serum selenium concentrations are associated with metabolic abnormalities in individuals with obesity and that sex differences exist.

Many studies have demonstrated that increasing dietary selenium levels through food processing techniques (29, 30) or supplementary selenium supplementation can confer cardiovascular health benefits. However, excessive selenium has adverse or even toxic effects on health (31). In the present study, we employed a larger sample size and a cross-sectional design using data from the 2011-2018 NHANES to ascertain the relationship between serum selenium concentrations and the progression of MHO to MUO in individuals with obesity. The results revealed that the weighted mean selenium concentration of the study participants was 2.46 μM (min: 1.33 µM or 105 µg/L, max:4.09 µM or 322 µg/L), which is above the average concentration for residents of the United States (approximately 1.74 µM or 137 µg/L) (32, 33) and Europe (1.09 µM or 85.8 µg/L) (34). Moreover, weighted mean selenium concentration was higher in the MUO group than in the MHO group. Furthermore, after adjusting for all covariates, the results of the generalized weighted regression analysis indicated that elevated serum selenium was associated with an increased risk of metabolic abnormalities in obese individuals. The presence of elevated serum selenium concentrations may induce dysregulation of metabolic homeostasis *in vivo*, thereby accelerating the transition from MHO to MUO.

The results of our investigation provide novel insights into the relationship between selenium and metabolic homeostasis. First, we observed that there were sex-based differences in the correlation between serum selenium concentrations and the risk of metabolic abnormalities. In males, the serum selenium concentration exhibited an inverted U-shaped correlation with metabolic abnormalities, whereas in females, the correlation between the selenium concentration and metabolic dysfunction was linear.

In the present study population, the weighted mean concentration of serum selenium was 2.50 µM in males, whereas it was 2.43 µM in females (*P*<0.001). This finding is consistent with the results of previous studies that reported higher serum selenium concentrations in males than in females (35, 36). Furthermore, no significant difference in the weighted mean concentration of selenium was detected between the MHO and MUO groups (2.50 *vs.* 2.51 µM, *P*=0.699) in males, but a significant difference was detected in females (2.41 *vs.* 2.46 µM, *P*=0.003). We postulate that the discrepancies in outcomes between the sexes may be attributed to variations in selenium uptake by sex organs or hormones. For example, selenium is retained in the testes of males (37), whereas there is no evidence that selenium uptake or retention by the female reproductive system is significant (38). Consequently, when selenium intake is excessive and results in increased selenium uptake in the body, alterations in metabolic health status are more pronounced in females.

Second, the most prominent symptom of obesity is abnormal serum lipid metabolism. All participants in this study were diagnosed with obesity on the basis of their BMI. Accordingly, the weighted prevalence of hyperlipidemia was not significantly different between the MHO (78.75%) and MUO (79.03%) groups. A comparison of the T1-3 groups according to the classification of selenium concentrations revealed that TG and TC concentrations were significantly higher in the T3 subgroup, whereas the opposite was observed for HDL-C. In females, elevated serum selenium concentrations above the saturation threshold were associated with significantly higher TC (weighted β=0.54, 95% CI= 0.32 - 0.76; *P*=0.000) and TG (weighted β=0.51, 95% CI= 0.27 - 0.75; *P*=0.000) concentrations. These findings indicate that alterations in the serum lipid profile represent a crucial phenomenon through which excess selenium *in vivo* induces metabolic remodeling in females.

Third, the majority of previous studies have indicated that there is no statistically significant correlation between selenium levels and hypertension. Nevertheless, a limited number of studies have indicated that selenium may contribute to the prevention of hypertension. The results of our study demonstrated a statistically significant discrepancy in the weighted incidence of hypertension between MHO (31.85%) and MUO (69.01%). Furthermore, the impact of the serum selenium concentration on blood pressure above and below the threshold interval was calculated. Interestingly, male participants exhibited heightened sensitivity to lower serum selenium concentrations. For example, elevated serum selenium concentrations have been demonstrated to increase the risk of elevated DBP when serum selenium concentrations are less than 2.82 µM. Conversely, when serum selenium exceeded the threshold, elevated selenium was accompanied by a decrease in SBP (weighted β: -10.70, 95% CI: -17.83 - 3.57, P=0.005). Notably, this study exclusively examined the influence of selenium concentrations below the saturation threshold on blood pressure in males. Nevertheless, selenium plays an essential role in numerous physiological processes within the body and must be maintained at optimal levels to sustain normal biological function. Accordingly, further research is required to determine the selenium threshold that causes elevated blood pressure.

This study has several potential limitations. First, the current study examined only the effects of selenium on metabolic abnormalities and did not consider the potential influence of other minerals or micronutrients. Second, all of the study participants were obese, thus the study did not control for the effects of diet and genetics on the outcome. Third, as our study was conducted exclusively with adult participants in the United States, the findings may be limited in terms of their generalizability. Nevertheless, the study also has several strengths. First, the sample size of the present study was sufficiently large to permit a clear distinction between MHOs and MUOs. Second, the NHANES-recommended data weighting and merging method was employed, thereby facilitating a more comprehensive analysis of the potential role of selenium in the metabolic transition from healthy to unhealthy.

In conclusion, the results of our study indicate that serum selenium plays a significant role in the metabolic remodeling observed in participants with MHO. Elevated serum selenium concentrations have been demonstrated to increase the risk of the conversion of MHO to MUO, and this risk appears to differ by sex. In males, an inverted U-shaped correlation pattern was demonstrated, as evidenced by alterations in blood pressure. In females, a linear correlation was observed, with serum selenium concentrations exceeding a threshold increasing the likelihood of hyperlipidemia and facilitating the conversion of MHO to MUO by modulating serum lipid alterations.

## Data Availability

Publicly available datasets were analyzed in this study. This data can be found here: https://wwwn.cdc.gov/Nchs/Nhanes/2011-2012/PBCD_G.htm; https://wwwn.cdc.gov/Nchs/Nhanes/2013-2014/PBCD_H.htm; https://wwwn.cdc.gov/Nchs/Nhanes/2015-2016/PBCD_I.htm; https://wwwn.cdc.gov/Nchs/Nhanes/2017-2018/PBCD_J.htm.
