# Supplementary material for: Serum selenium accelerates the development of metabolic disorders in a metabolically healthy obese U.S. population: a retrospective cross-sectional analysis of a population-based study from the NHANES (2011-2018)
Source: Front Immunol. 2024 Aug 29;15:1398299. doi: 10.3389/fimmu.2024.1398299 (PMC11390505; doi:10.3389/fimmu.2024.1398299)
Supplement: Supplementary file 1 [file DataSheet1.docx]

**Supplementary Table 1** Weighted characteristics of the study participants

| **Characteristic** | **MHO** | **MUO** | ***P* value** |
| --- | --- | --- | --- |
| **Selenium** (µmol/L) | 2.45 (2.43, 2.47) | 2.48 (2.46, 2.51) | 0.006 |
| **Age** (Years) | 43.35 (42.50, 44.20) | 53.89 (53.14, 54.64) | <0.001 |
| **Sex** (%) |  |  | <0.001 |
| Male  Female | 41.00 (38.99, 43.03)  59.00 (56.97, 61.01) | 52.24 (49.42, 55.05)  47.76 (44.95, 50.58) |  |
| **Race/Ethnicity** (%) |  |  | <0.001 |
| Non-Hispanic White | 60.50 (55.95, 64.86) | 66.71 (62.67, 70.51) |  |
| Non-Hispanic Black | 14.18 (11.54, 17.29) | 13.78 (11.28, 16.73) |  |
| Non-Hispanic Asian | 2.13 (1.68, 2.69) | 1.68 (1.27, 2.20) |  |
| Mexican American | 11.74 (9.33, 14.69) | 9.14 (7.23, 11.48) |  |
| Others | 11.45 (9.91, 13.20) | 8.70 (7.26, 10.40) |  |
| **Marital Status** (%) |  |  | <0.001 |
| Married/Living with partner | 62.30 (59.87, 64.67) | 62.77 (59.80, 65.64) |  |
| Widowed/Divorced/Separated | 17.06 (15.38, 18.89) | 23.82 (21.52, 26.30) |  |
| Never married | 20.63 (18.14, 23.36) | 13.38 (11.71, 15.23) |  |
| **Education** (%) |  |  | 0.510 |
| Collage and above | 59.53 (56.13, 62.84) | 60.77 (58.09, 63.40) |  |
| High school | 26.64 (23.93, 29.54) | 24.70 (22.56, 26.98) |  |
| Less than high school | 13.79 (12.04, 15.75) | 14.52 (12.68, 16.57) |  |
| **Family Size** (%) |  |  | <0.001 |
| 1-3 people | 61.52 (58.77, 64.21) | 72.93 (70.50, 75.23) |  |
| 4-6 people | 34.94 (32.30, 37.66) | 24.86 (22.66, 27.20) |  |
| More than 7 people | 3.54 (2.65, 4.70) | 2.21 (1.72, 2.83) |  |
| **Family income** (%) |  |  | 0.792 |
| < $45,000 | 44.40 (41.74, 47.09) | 43.32 (39.90, 46.82) |  |
| $45,000-$99,999 | 31.51 (29.06, 34.07) | 32.25 (28.25, 36.53) |  |
| ≥$100,000 | 19.27 (16.74, 22.08) | 20.24 (17.36, 23.47) |  |
| **Smoking** (%) |  |  | <0.001 |
| Never | 57.95 (55.01, 60.82) | 53.43 (50.31, 56.52) |  |
| Former | 23.62 (20.99, 26.47) | 31.94 (29.32, 34.67) |  |
| Now | 18.40 (16.40, 20.58) | 14.61 (12.98, 16.40) |  |
| **Alcohol user** (%) |  |  | 0.003 |
| Never | 8.74 (7.96, 9.93) | 8.46 (7.20, 9.91) |  |
| Former | 9.90 (8.50, 11.50) | 10.43 (8.99, 12.07) |  |
| Mild | 30.54 (27.93, 33.29) | 35.49 (32.85, 38.22) |  |
| Moderate | 15.79 (13.80, 18.01) | 16.81 (14.80, 19.02) |  |
| Heavy | 23.08 (21.16, 25.12) | 17.51 (15.26, 20.01) |  |
| **Work Activity** (%) |  |  | 0.563 |
| No | 51.71 (48.58, 54.82) | 52.87 (50.08, 55.65) |  |
| Yes | 48.29 (45.18, 51.42) | 47.13 (44.35, 49.92) |  |
| **Recreational Activity** (%) |  |  | 0.002 |
| No | 50.27 (47.38, 53.16) | 56.41 (53.18, 59.60) |  |
| Yes | 49.73 (46.84, 52.62) | 43.59 (40.40, 46.82) |  |
| BMI (Kg/m^2^) | 35.99 (35.68, 36.30) | 36.30 (35.95, 36.64) | 0.156 |
| Height (cm) | 167.2 (166.7, 167.6) | 168.8 (168.2, 169.4) | 0.000 |
| Weight (kg) | 100.7 (99.8, 101.6) | 103.7 (102.6, 104.7) | 0.000 |
| Waist circumference (cm) | 113.4 (112.7, 114.2) | 117.1 (116.3, 117.9) | <0.001 |
| Glucose (mM) | 5.57 (5.48, 5.66) | 6.26 (6.15, 6.36) | <0.001 |
| HBA1C (%) | 5.72 (5.67, 5.77) | 6.05 (5.99, 6.11) | <0.001 |
| TC (mM) | 4.90 (4.84, 4.95) | 5.08 (5.01, 5.15) | <0.001 |
| TG (mM) | 2.05 (1.98, 2.13) | 1.93 (1.85, 2.02) | 0.015 |
| HDL-C (mM) | 1.19 (1.17, 1.21) | 1.31 (1.29, 1.33) | <0.001 |
| LDL-C (mM) | 2.83 (2.74, 2.92) | 3.00 (2.94, 3.06) | 0.005 |
| SBP (mmHg) | 120 (119, 121) | 131 (130, 132) | <0.001 |
| DBP (mmHg) | 71 (71, 72) | 75 (74, 76) | <0.001 |

**Notes.**

Data in the table: For continuous variables: survey-weighted means (95% CI), and *P*-value were obtained via survey-weighted linear regression (svyglm); For categorical variables: survey-weighted percentages (95% CI), and *P*-value was by survey-weighted chi-square test (svytable).

**Abbreviations**: Body mass index, BMI; Glycohemoglobin, HBA1C; Total Cholesterol, TC; Triglyceride, TG; HDL cholesterol, HDL.C; LDL cholesterol, LDL.C; Systolic blood pressure, SBP; Diastolic blood pressure, DBP, metabolically healthy obesity, MHO; metabolically unhealthy obesity, MUO.

**Supplementary Table 2** Population with metabolic abnormalities in study participants with MHO and MUO

|  | **MHO** | **MUO** | ***P* value** |
| --- | --- | --- | --- |
| **Hypertension** |  |  | <0.001 |
| No | 68.15 (65.44, 70.73) | 30.99 (28.53, 33.57) |  |
| Yes | 31.85 (29.27, 34.56) | 69.01 (66.43, 71.47) |  |
| **Hyperlipidemia** |  |  | 0.862 |
| No | 21.25 (19.00, 23.68) | 20.97 (18.72, 23.41) |  |
| Yes | 78.75 (76.32, 81.00) | 79.03 (76.59, 81.28) |  |
| **DM** |  |  | <0.001 |
| No | 79.91 (78.00, 81.69) | 52.09 (49.08, 55.09) |  |
| Yes | 16.27 (14.62, 18.06) | 31.56 (29.07, 34.16) |  |
| Other | 3.82 (2.92, 5.00) | 16.35 (14.37, 18.54) |  |
| **CHD** |  |  | 0.002 |
| No | 96.92 (95.65, 97.83) | 94.62 (92.98, 95.90) |  |
| Yes | 3.08 (2.17, 4.35) | 5.38 (4.10, 7.02) |  |
| **Stroke** |  |  | 0.012 |
| No | 97.34 (96.46, 98.01) | 95.82 (94.82, 96.64) |  |
| Yes | 2.66 (1.99, 3.54) | 4.18 (3.36, 5.18) |  |

Notes.

For categorical variables: survey-weighted percentage (95% CI), *P*-value was by survey-weighted Chi-square test (svytable).

**Abbreviations**: coronary heart disease, CHD; diabetes mellitus, DM; metabolically healthy obesity, MHO; metabolically unhealthy obesity, MUO.
